# A Broadband Transmitarray Antenna Using a Metasurface-Based Element for Millimeter-Wave Applications

**DOI:** 10.3390/mi15030383

**Published:** 2024-03-13

**Authors:** Yue Cao, Miaojuan Zhang, Chong Fan, Jian-Xin Chen

**Affiliations:** 1 School of Information Science and Technology, Nantong University, Nantong 226019, China; 2330310047@stmail.ntu.edu.cn (M.Z.); jjxchen@hotmail.com (J.-X.C.); 2 Guangdong Provincial Key Laboratory of Millimeter-Wave and Terahertz, School of Electronic and Information Engineering, South China University of Technology, Guangzhou 510641, China; 3 Nanjing Electronic Devices Institute, Nanjing 210016, China; fan_chong@163.com

**Keywords:** transmitarray antenna, metasurface, millimeter-wave, wideband

## Abstract

In this manuscript, a broadband transmitarray antenna (TA) using a metasurface-based element is presented for millimeter-wave communication applications. The metasurface-based TA element adopts a receiver–transmitter configuration: metasurfaces are applied as the receiver and transmitter, and slot-coupled differentially fed striplines are used as the phase compensation. The designed TA element achieves good transmission performance with a more than 360° transmission phase shift range and less than 1-dB transmission insertion loss within a wide frequency range. To verify the proposed TA, a prototype is fabricated based on the conventional printed circuit board (PCB) process, and a pyramid horn is designed as the source. The measured results show that the proposed TA with the differential feed network presents a 1-dB gain bandwidth of 26.2% from 23.5 to 30.5 GHz and a peak gain of 24.5 dBi. The designed TA is a promising alternative for millimeter-wave communications applications because of its high gain, broad bandwidth, low costs, and convenient integration with other circuits.

## 1. Introduction

As one kind of air-fed array antennas, transmitarray antennas (TAs) have attracted more and more attention on account of their good characteristics, including their high gain, lack of feeding aperture blockage, and lack of a complex feed network. Therefore, TAs are promising alternatives for millimeter-wave (mm-wave) applications [1,2]. A classical TA consists of a source antenna and a transmission array. The source antenna is usually located at the focal point of the TA. The transmission array is composed of many printed units and is distanced from the source antenna. Each unit incorporates a high-efficiency transmission magnitude and transmission phase to form the desired planar outgoing wavefront. Therefore, a focused high-gain beam can be obtained. However, the traditional TA units usually have a narrow transmission bandwidth [3]. In addition, the spatial path from the source antenna to each unit located in the different locations of the transmission array aperture is frequency-dependent. Consequently, TAs are subjected to a narrow bandwidth.

Recently, many reported works have paid much attention to the bandwidth improvement of transmitarray antennas, which can be mainly grouped into two types of techniques. The first technique uses multilayer frequency selective surfaces (FSS) as spatial filters to realize the wideband performance. Instead of a single-layer structure, multilayer FSS structures separated by a substrate or air gap are adopted as the TA’s element, such as four-layer double square rings [4], triple-layer spiral dipole elements [5], triple-layer elements based on cross-slots, double square rings [6] and three-layer multi-resonant element [7]. The TAs based on these multilayer FSS elements in [4,5,6,7] achieved 1-dB gain bandwidths of 7.5%, 9%, 16.8%, and 37.3%, respectively. The elements in [4,5,6,7] were separated by air gaps, which increased the complexity of the assembly in practical applications. Additionally, the elements based on multilayer FSS structures have more layers, which results in a high profile. In [8], a three-layer TA element without air gaps was proposed. The adjacent dielectric substrates were bonded using a bonding film to lower the element profile. In [9], an FSS unit with only two metal layers was proposed to achieve a low-profile TA antenna. The TA antenna obtained a 3-dB gain bandwidth of 15%. In addition, two low-profile double-layer TA elements were designed in [10,11]. Four metal vias were adopted to augment the transmission magnitude. A 1-dB gain bandwidth of 5.9% and an aperture efficiency of 40% were achieved in [10]. In [11], the TA had a 1-dB gain bandwidth of 9% and an aperture efficiency of 51.4%. However, the phase compensation of the elements based on multilayer FSS is related to the transmission characteristic and cannot be adjusted independently, so the bandwidth improvement is limited.

The other type of technique employs a receiver–transmitter configuration, which generally consists of a receiving plane, an interconnected phase compensation plane, and a radiating plane [12,13]. The receiving plane is responsible for capturing the incident electromagnetic wave from the source antenna. The phase compensation plane modulates the phase of the received electromagnetic wave to the desired wavefront. Finally, the radiating plane emits the modulated electromagnetic wave into the free space. Based on this configuration, a TA has the performances of a 3.6-dB average insertion loss, and a 10% 3.4-dB gain bandwidth was realized in [14], which used a single-patch antenna as the receiving plane and radiating plane, and a balanced bridged-T phase shifter as the phase compensation plane. In [15], an element based on a tightly coupled dipole array was reported. In this design, the true-time delay (TTD) line was adopted as the TA’s transmission phase compensation plane, which helped to significantly increase the TA’s bandwidth. Moreover, in [16], a lens unit cell based on fourth-order miniaturized element frequency selective surfaces was proposed. The proposed lens provided a frequency-independent time delay within the frequency band of interest and achieved a 38% gain bandwidth for a 3-dB gain drop. Although some efforts were undertaken to improve the phase compensation range, the increase in bandwidth is still limited because of the inherent narrow bandwidth of the receiving and radiating plane element. Furthermore, the bandwidth of the transmission magnitude still needs to be improved.

In this design, a TA with high gain and broad bandwidth using metasurface-based elements for a millimeter-wave application was realized. The metasurface was applied as the receiver and transmitter and was responsible for the transmission amplitude bandwidth. The pair of striplines was used as the phase compensation. Different from the traditional TA element, the transmission amplitude and phase compensation of the proposed metasurface-based TA element were adjusted independently within a wide frequency range. The organization of the manuscript is as follows. Firstly, the proposal and evolution of the proposed TA element are introduced in detail. Meanwhile, the metasurface-based TA element adopting the receiver–transmitter configuration is introduced, which consists of a metasurface antenna as the receiver array and transmitter array, together with different lengths of striplines as the phase delay array. Then, aiming to increase the bandwidth performance, research on the transmission amplitude and transmission phase response of the proposed TA element as a function of frequency is presented in detail. Finally, a prototype and the metal feeding pyramid horn are fabricated and measured, which shows the proposed transmitarray antenna has a 26.2% bandwidth for a 1-dB gain reduction and a peak gain of 24.5 dBi.

## 2. Design and Analysis of the TA Element

### 2.1. The Proposal and Evolution of the TA Element

In order to realize a broadband TA, the transmission bandwidth of the TA element is very critical. In this section, the proposal and evolution of the TA element are introduced in detail. First of all, a wideband single-polarized metasurface antenna fed by a stripline is designed in Section 2.1.1. Then, a dual-polarized metasurface antenna fed by striplines is proposed in Section 2.1.2. In the end, the evolution of the TA element based on the previous metasurface antenna is displayed in Section 2.1.3.

#### 2.1.1. Single Linearly Polarized Metasurface Antenna Fed by a Stripline

**Figure 1:**
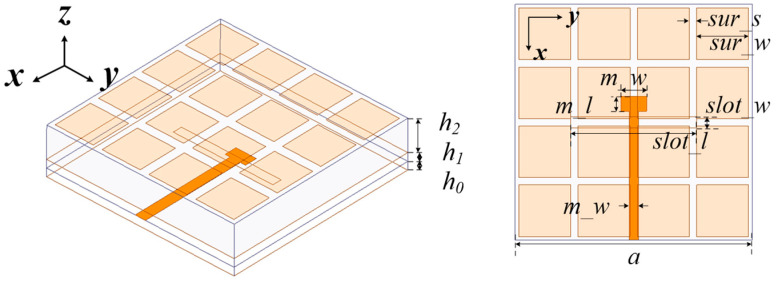
Structure of the single linear-polarized metasurface antenna where *slot_w* = 0.3 mm, *slot_l* = 3.2 mm, *m_w* = 0.7 mm, *m_l* = 0.4 mm, *sur_s* = 1.4 mm, *sur_w* = 0.2 mm, *m_w* = 0.25 mm, and *a* = 6.4 mm.

Recently, metasurface (MS) antennas [17,18,19] with wide-band and low-profile performances have received more and more attention. In this work, a 4 × 4 square patch metasurface antenna fed by striplines was designed. The structure of the metasurface antenna fed by striplines is exhibited in Figure 1. It was composed of three substrate layers and four metal layers. The three substrates of the metasurface antenna were all Rogers Duroid 4003, with a relative dielectric constant of 3.55 and a loss tangent of 0.0027. The thickness of the upper substrate was *h*_2_, with a value of 0.762 mm. The thickness of the middle substrate was *h*_1_, with a value of 0.254 mm. Additionally, *h*_0_ was the thickness of the bottom substrate, whose value was the same as that of the middle substrate. Different from most of the conventional microstrip feeding metasurface antennas, the stripline was adopted as the feeding line. There are two reasons for using striplines. The first is that the microstrip line is a semi-open transmission line structure that propagates the primary mode, which is a quasi-TEM wave. However, as a fully enclosed transmission line, the stripline transmits TEM mode electromagnetic waves, and its upper and lower sides have a ground layer. This brings great advantages, such as good signal shielding and small transmission loss. In addition, because of the semi-open structure of the microstrip line, it can generally only be placed on the top or bottom layer of the antenna, and the signal can only be connected in a single direction. As a result, the electromagnetic wave can only be radiated and transmitted in one single direction, which limits the degree of design freedom. However, due to the existence of two ground layers, the circuit based on the stripline can be expanded in both the upper and lower dimensions, so there is greater design freedom than the microstrip.

**Figure 2:**
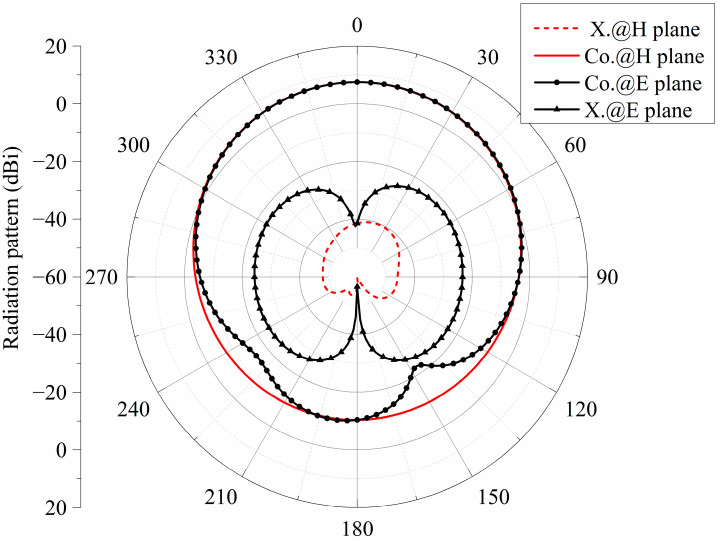
Radiation pattern of the single linear-polarized metasurface antenna.

**Figure 3:**
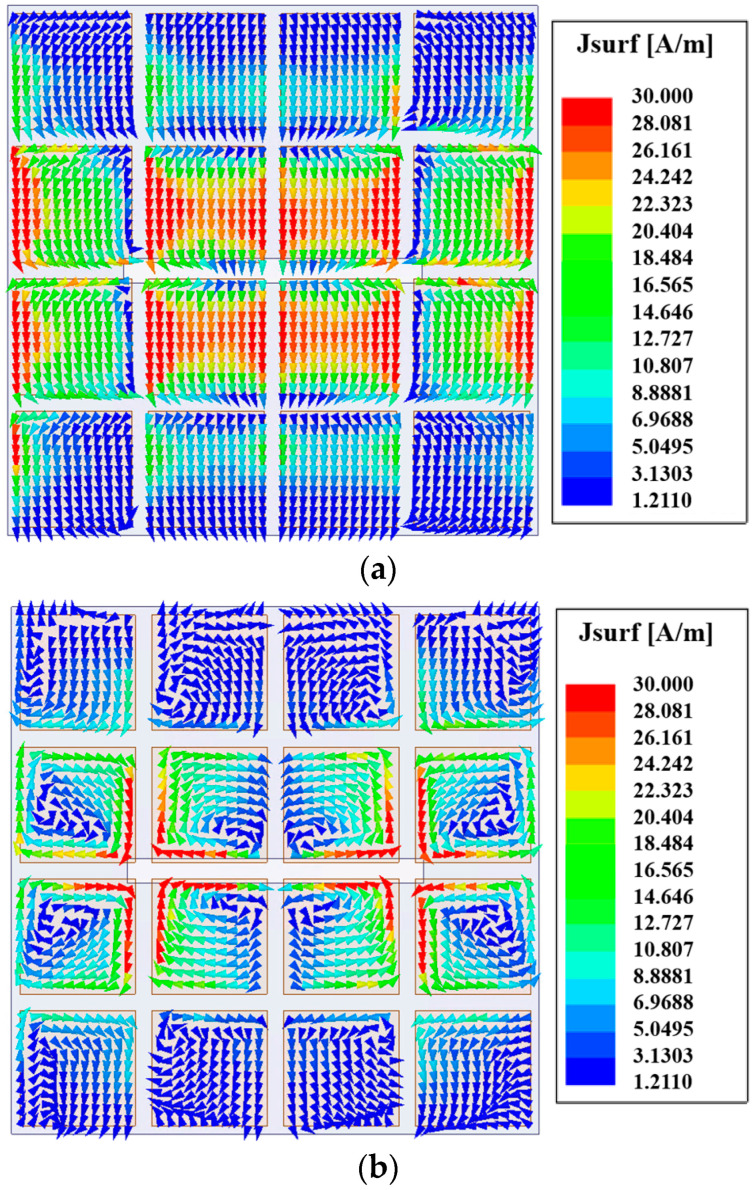
Surface current distribution of the metasurface at (**a**) 23.2 GHz and (**b**) 25.9 GHz.

Figure 2 shows the radiation patterns of the E- and H-planes of the antenna at the frequency of 24.6 GHz. It can be seen from the figure that the gain is 7 dBi. The main polarization pattern of the H-plane was symmetrical, and the cross-polarization level of the H-plane was less than −47 dB. However, the symmetry of the main polarization pattern of the E-plane was not very good. The cross-polarization level of the E-plane was slightly higher because of the asymmetry of the structure along the *x*-axis. Figure 3 is the surface current distribution of the square patch layer of the antenna at two resonant frequency points (23.2 GHz and 25.9 GHz). As seen from the figure, the surface current of the middle two rows of the square patch metasurface antenna was stronger, while the surface current of the top and bottom rows of the patch was weaker.

#### 2.1.2. Differential-Driven Metasurface Antenna Fed by Striplines

**Figure 4:**
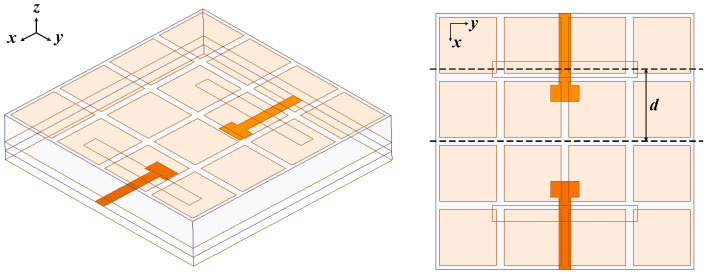
Geometry of the differential-driven metasurface antenna.

In order to improve the E-plane cross-polarization of the single linearly polarized metasurface antenna, a differential-driven metasurface antenna fed by striplines is proposed in this section. The geometry of the differential-driven metasurface antenna is shown in Figure 4. The substrates and the sizes of the square patches and the striplines were the same as that in Section 2.1.1. The difference was that the coupling slot of the antenna was improved from one single slot in the middle of the antenna to double slots. The distance from the center symmetry line is *d*, with a value of 1.7 mm. Moreover, the feeding lines also increased to two. In order to render the structure symmetrical and avoid the crossover of the pair of striplines, the pair of striplines was designed back-to-back. In addition, in order to ensure that the electric fields transmitted to the two coupled slots were in constant amplitude and in phase, the two gaps were fed in a constant amplitude and in phase by providing a pair of back-to-back differential signals.

**Figure 5:**
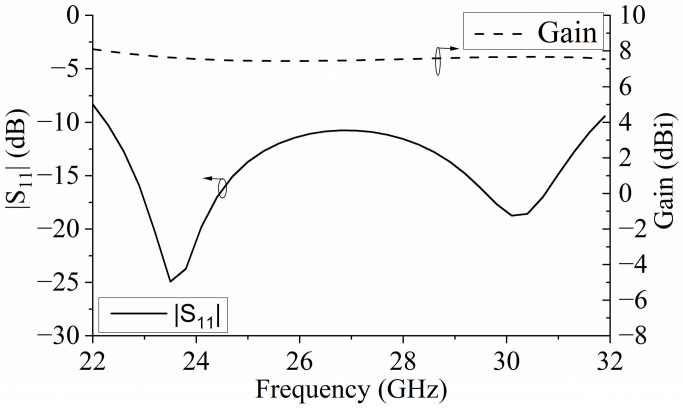
S parameter and gain of the differential-driven metasurface antenna.

**Figure 6:**
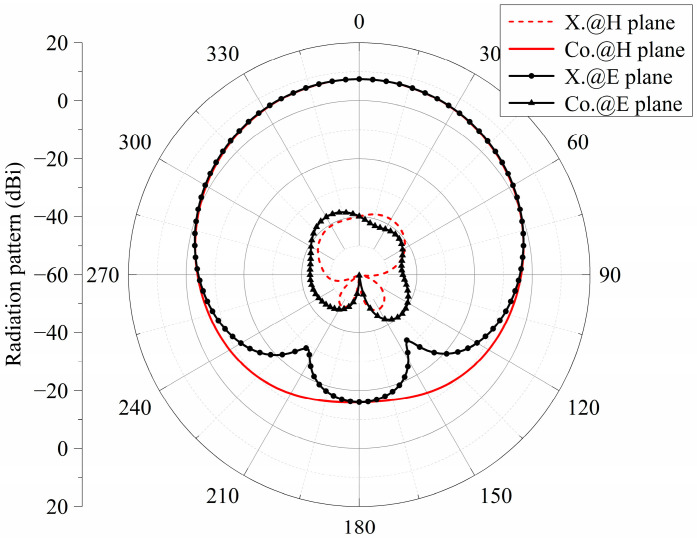
Radiation pattern of the differential-driven metasurface antenna.

**Figure 7:**
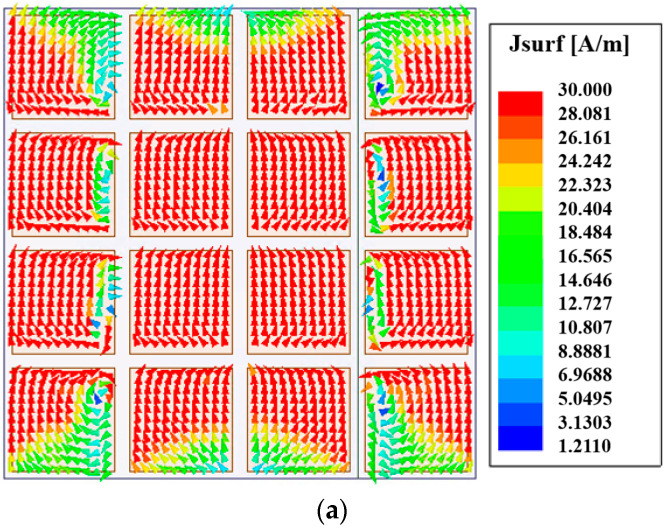

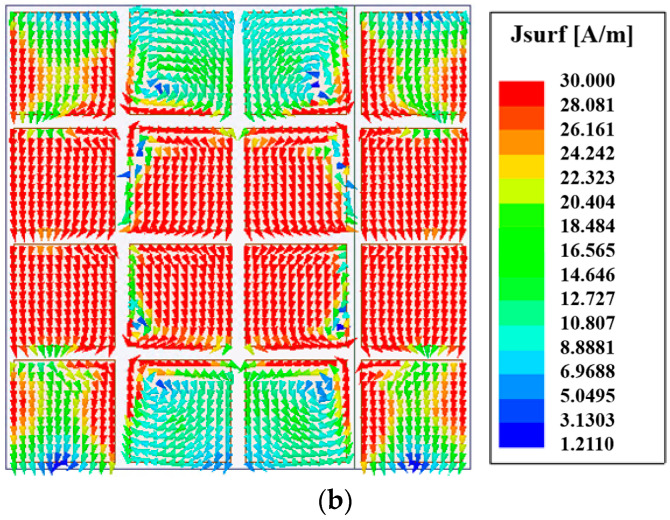
Surface current distribution of the metasurface at two frequency points: (**a**) 23.5 GHz and (**b**) 30.1 GHz.

The simulated S parameters and gain curves are shown in Figure 5 under the radiation boundary condition. The |S_11_| in Figure 5 was calculated using the formulas in [20]. As seen, the |S_11_| is lower than −10 dB within the frequency range of 22.2 to 31.8 GHz. The impedance bandwidth of −10 dB was 35.6%. In the range of 22.2–31.8 GHz, the value of the gain was basically stable at 7.5 dBi, which increased the gain by 0.5 dBi compared with the single-polarized metasurface antenna in Section 2.1.1. Figure 6 shows the radiation patterns of the E- and H-planes of the metasurface antenna at the central frequency of 27 GHz. Compared with the single-polarized metasurface antenna proposed in the previous section, the radiation patterns of the E- and H-planes of the antenna are symmetrical. With the introduction of a pair of differential feed striplines, the antenna was symmetrical on the E-plane, and the cross-polarization level of the E-plane was greatly improved. In addition, the front-to-back ratio of the antenna radiation pattern was also improved to 23.5 dB, which is 8 dB higher than the front-to-back ratio of 15.5 dB in the previous section. Figure 7 is the surface current distribution diagram of the square patch layer of the differential double-slot coupled feed metasurface antenna at the two resonant frequency points of 23.5 GHz and 30.1 GHz. It can be seen from the diagram that compared with the single-slot coupled feed metasurface antenna, the metasurface antenna coupled to the differential double-slot can not only excite the middle two rows of square patches but also effectively excite the upper and lower rows of patches. The surface current of the entire 4 × 4 square patch antenna was more uniform, and the current intensity was stronger, which helped to improve the antenna gain, further supporting the conclusion that the antenna gain was increased by 0.5 dBi as shown in Figure 6.

#### 2.1.3. Structure of the TA Element

**Figure 8:**
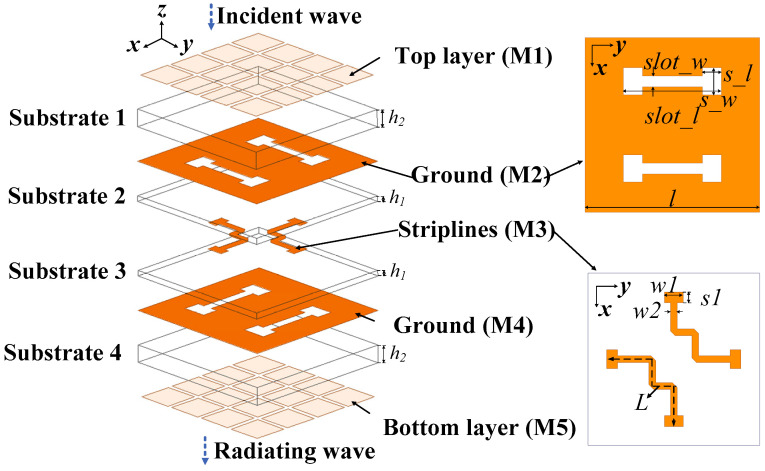
Structure of the proposed metasurface-based element where *slot_w* = 0.3 mm, *s_l* = 3.2 mm, *s_l* = 0.7 mm, *s_w* = 0.4 mm, *w*1 = 0.7 mm, *w*2 = 0.25 mm, *s*1 = 0.4 mm, and *l* = 6.4 mm.

Inspired by the MS antennas above, an MS-based TA element is proposed in this section. The geometry and detailed sizes of the MS-based TA element are illustrated in Figure 8. The TA element was a multi-layer structure consisting of four layers of substrate and five layers of metal. The proposed TA element was based on the receiver–transmitter configuration [12]. Substrates 1, 2, 3, and 4 utilized the same substrates (Rogers Duroid 4003, *ε_r_* = 3.55, *tanδ* = 0.0027) with a thickness of *h*_2_ = 0.762 mm and *h*_1_ = 0.254 mm. The top metal layer (M1) was composed of 4 × 4 square sub-patches. M1, substrate 1, and the ground plane (M2) were a typical metasurface antenna, which acted as the receiving plane. Analogously, the bottom layer (M5), substrate 4, and ground plane (M4) acted as the radiating plane. Moreover, the middle substrate 2 and substrate 3, together with the M2, M4, and the striplines (M3), acted as the phase compensation plane. It should be noted that the striplines on M3 were one type of layout (type 1) but other types of stripline layouts can also be used in order to obtain more than 360° phase delay. In order to achieve the function of receiving and transmitting waves, the receiving and transmitting planes of the element needed to be placed orthogonally. Therefore, the direction of the coupling slots on M2 and M4 was orthogonal, and the two pairs of striplines on M3 were also orthogonal. Furthermore, to avoid the two pairs of striplines crossing in the middle of the element, the striplines were bent by 90° three times. In addition, the shape of the coupling slots on the ground plane (M2 and M4) was changed from a rectangle (Figure 1 and Figure 4) to a dumbbell (Figure 8) for a better match.

As shown in Figure 8, the TA element works as follows. It is assumed that the electromagnetic wave propagates in the positive direction of the *z*-axis to the negative direction of the *z*-axis. When the incident wave along the *x*-axis direction of the polarization direction of the electric field is incident on the upper receiver plane (M1) of the TA element, two coupling gaps in the upper ground layer (M2) couple the energy to the striplines of the phase delay line layer (M3). Because the stripline is bent by 90°, the transmission direction of the electromagnetic wave also rotates by 90°. When the electromagnetic wave is transmitted to the other end of the striplines, the polarization direction of the electric field is reversed by 90° and becomes along the *y*-axis direction. At this time, it matches the coupling gap along the *x*-axis in the lower ground plane (M4) and radiates the electromagnetic wave through the lower transmitter plane (M5).

In short, when the layout of the coupling gap and transmission line is designed properly, the metasurface antenna can be used in the application of the TA element. In order to receive and radiate the incident electromagnetic waves efficiently in the wide band range, the metasurface antenna located on the top layer and bottom layer could be designed rationally. In addition, by adjusting the length (*L*) of the delay lines, an up-to-400° phase shift range can be obtained. It is worth mentioning that, unlike the traditional method of adjusting the delay phase by changing the size of the TA element, there is no need to change the size of the proposed metasurface-based TA element. The phase delay can be obtained by changing the length of the transmission line, which does not affect the resonant frequency of the TA element. That is to say, the transmission and phase compensation of the proposed TA element are realized independently and do not interfere with each other.

### 2.2. Performance and Analysis of the TA Element

**Figure 9:**
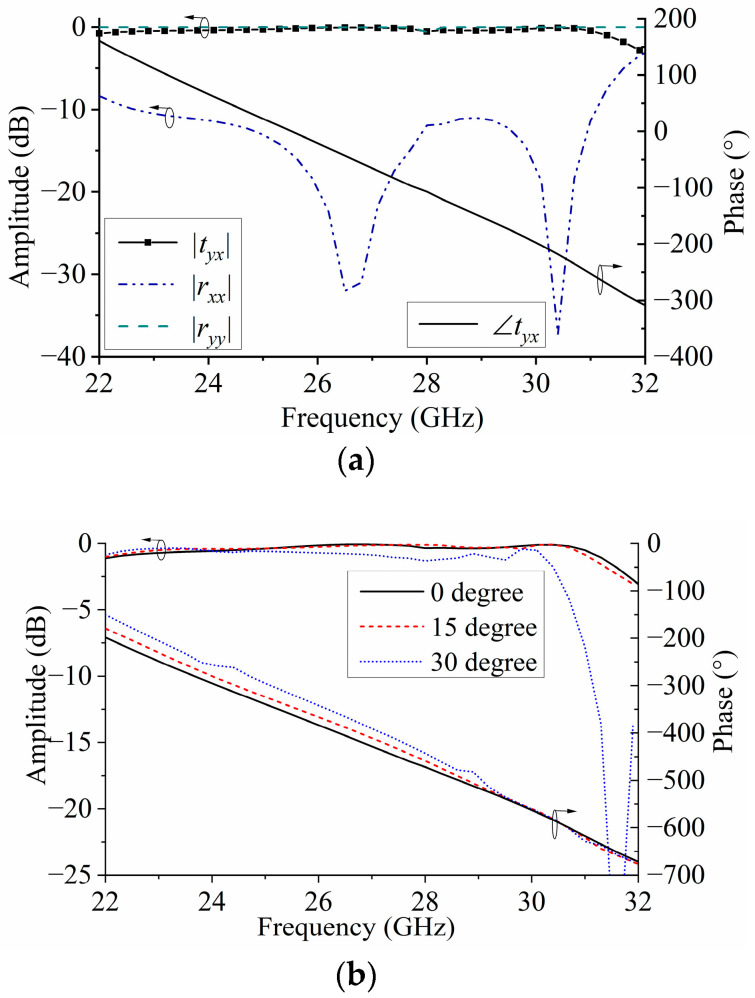
Simulated transmission amplitude and phase responses at a vertical incidence (**a**) and oblique incidence (**b**).

The commercial simulation software HFSS 2022 R2 was adopted to analyze the performances of the proposed TA element. The simulated results are shown in Figure 9, where |*t_yx_*| is the transmission amplitude, defined as the ratio of the transmitted electric field amplitude in the *y*-polarization direction to the incident electric field amplitude in the *x*-polarization direction. Additionally, |*r_xx_*| is the reflection amplitude, defined as the ratio of the reflected electric field in the *x*-polarization direction to the incident electric field in the *x*-polarization direction. Moreover, |*r_yy_*| is the reflection coefficient in the *y*-polarization for a y-polarized wave, and ∠*t_yx_* represents the transmitted phase. As seen in Figure 9a, the transmission amplitude of |*t_yx_*| is close to 0 dB, and the reflection amplitude of |*r_xx_*| is very weak, which indicates that almost all the incident waves have passed through the TA element. Furthermore, the reflection amplitude of |*r_yy_*| is close to zero, which shows that the electromagnetic wave in the *y*-polarization direction cannot pass through the structure and is almost reflected. Moreover, the solid black curve representing the transmission phase is almost linear as a function of frequency. It indicates that the phase linearity of the element is good, and it is beneficial to improve the TA bandwidth. Figure 9b shows the transmission amplitude and phase at the oblique incidence angles of 10°, 20°, and 30°. It can be seen that for incidence angles less than 30°, the phase error of the unit is less than 10°, and the transmission amplitude error is less than 1.5 dB.

**Figure 10:**
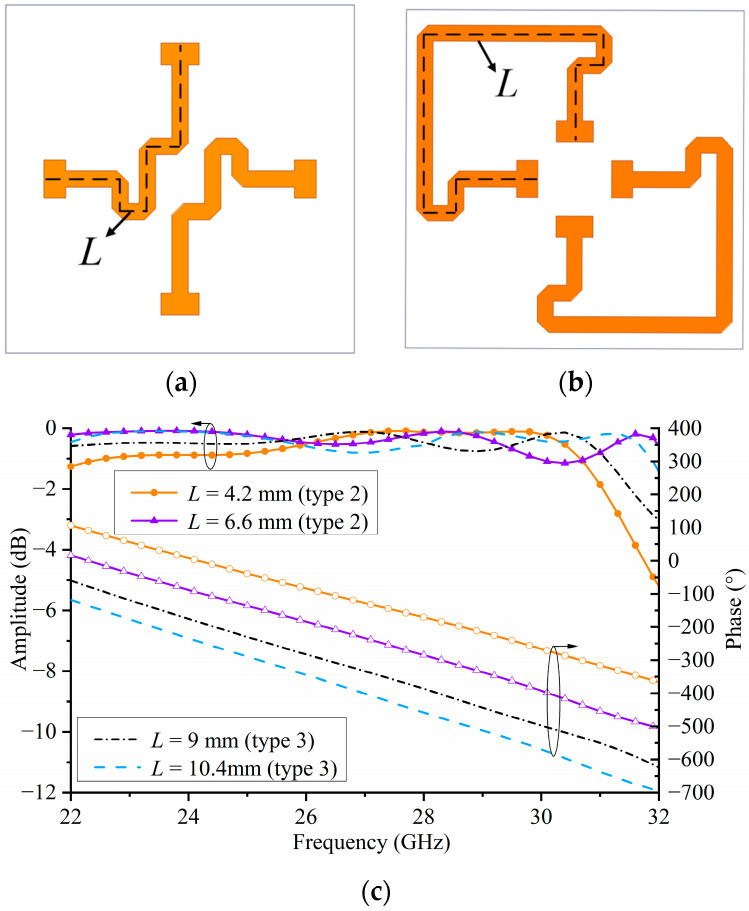
(**a**) Stripline arrangement on M3 of type 2, (**b**) stripline arrangement on M3 of type 3, and (**c**) simulated amplitude and phase responses of the proposed TA element in types 2 and 3.

In [15], the true-time delay (TTD) was introduced to achieve a linear response within a wide frequency range. By adjusting the length of the TTD lines on the phase delay plane other than changing the structure size on the receiver/transmitter plane to achieve the phase shift, thus the broadband performance of a TA is easier to achieve because the resonant frequency of the TA element is not changed. Because of the limited range of the phase shift of the stripline layout in Figure 8, the other two types of stripline layouts were introduced to achieve the full 360° transmission phase shift range. Moreover, the other two types of stripline layouts are shown in Figure 10a,b. By changing the length (*L*) of the striplines, the corresponding transmission magnitude and phase responses could be achieved, which is illustrated in Figure 10c. When the wave is under a normal incidence, the transmission losses of different lengths of striplines are small, achieving a 29.7% 1-dB insertion loss bandwidth. Dielectric loss, the radiation loss of the striplines, and the coupling mismatch of the interlayer gap are the main causes of the insertion loss. In future designs, the insertion loss of the element could be reduced by using lower-loss true-time delay lines and reducing the number of layers of the element. Moreover, it can be seen from Figure 10c that up to 400° linear phase compensation is obtained. The results also verify that adjusting the length of the TTD lines only changed the values of the phase responses and did not influence the resonance characteristics of the unit. Compared with the patch-based unit in [21], both simulation results of the proposed metasurface-based element and patch-based element [21] indicate a good agreement of the transmission performance. Moreover, the proposed metasurface-based element had a wider 1-dB insertion loss bandwidth because of the MS and TTD techniques.

In conclusion, the proposed TA element has some merits after the simulation analysis. Firstly, the proposed TA element achieves a high transmittance and low-loss transmission in the broadband range (from 22.4 to 30.2 GHz). Secondly, a phase compensation range of more than one cycle was obtained by adjusting the total lengths of the delay lines with ease. Thirdly, thanks to the independence of the receiving plane and the radiating plane, the proposed element had polarization selection and polarization rotation functions. In this work, the TA unit may twist the polarization direction of the incident wave from *x*-polarization to *y*-polarization and reflect the *y*-polarization incident wave.

## 3. Experiment Results and Discussion

**Figure 11:**
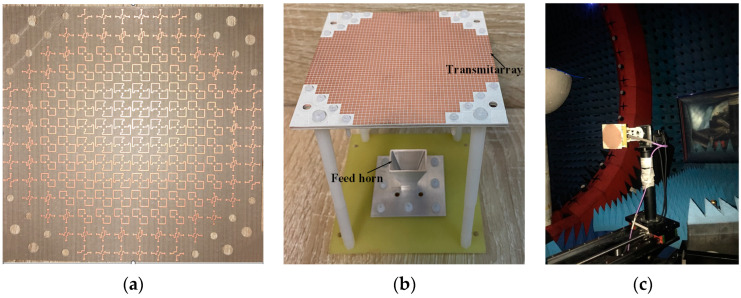
The photographs of the fabricated TA. (**a**) Phase delay layer of the TA. (**b**) 3D view of the TA. (**c**) Measurement environment of the TA.

Based on the above TA unit, one TA with a broad bandwidth is assembled for the millimeter-wave application. The center frequency of the TA was 27 GHz. The TA consisted of 156 elements with an aperture diameter of 44.8 × 44.8π mm^2^ (16π*λ*_0_^2^). A pyramid horn with a size of 21.5 × 14.5 mm^2^ and a height of 18.5 mm was located in the central position as the source. The simulated −10 dB beamwidth of the horn was 60°. Considering the aperture efficiency, the focal-to-diameter ratio *F*/*D* = 0.87 was applied. Thus, the height between the aperture of the horn and the transmission surface was 78 mm. Once the dimension of the TA was determined, the phase error values that needed to be compensated for each element at different positions on the TA could be calculated. For verification, the proposed broadband TA was fabricated using the conventional PCB process, and the photographs are shown in Figure 11. Figure 11a shows the desired phase compensation distribution. The phase values that need to be compensated for the edge part are small, and the phase values that need to be compensated for the middle position of the antenna are large. Figure 11b demonstrates the three-dimensional stereogram of the TA. The FR4 dielectric substrate, four nylon struts, and several screws were adopted to assemble the TA and the feed horn. As shown in Figure 11c, the experiments were carried out in an anechoic chamber using a far-field testing system. A cylindrical plastic fixture was used to fix the TA and the test system. In addition, a coaxial-to-waveguide adapter was needed to transmit the energy from the feeding cable of the test system to the feed horn of the measured TA.

**Figure 12:**
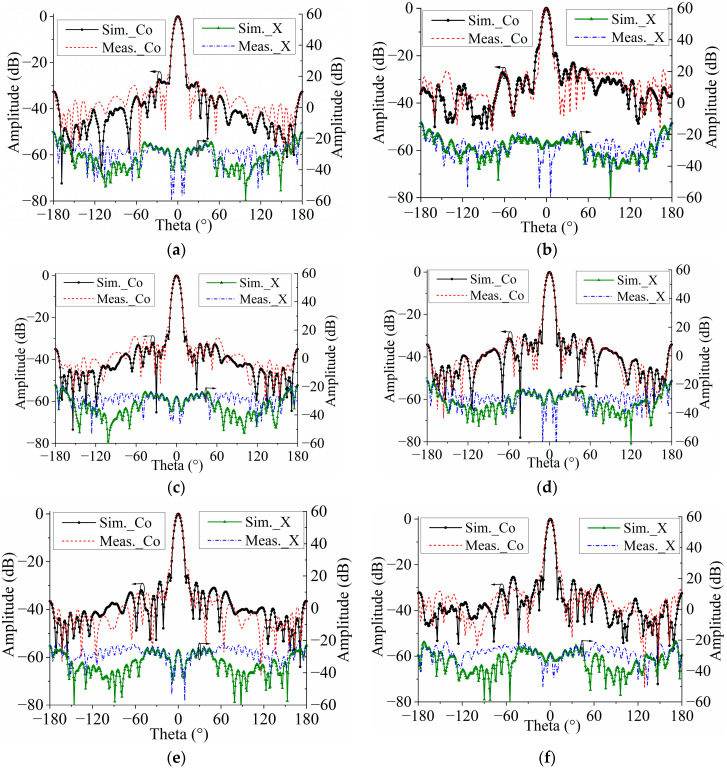
Simulated and measured normalized radiation patterns. (**a**) H-plane at 24 GHz. (**b**) E-plane at 24 GHz. (**c**) H-plane at 27 GHz. (**d**) E-plane at 27 GHz. (**e**) H-plane at 30 GHz. (**f**) E-plane at 30 GHz.

**Figure 13:**
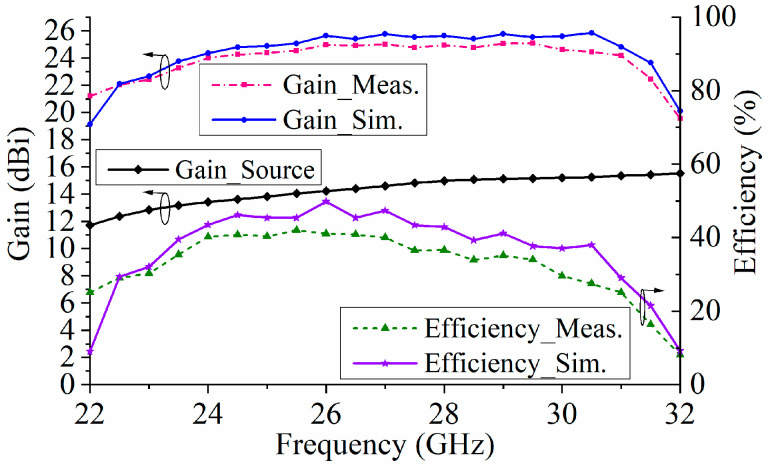
Simulated gain curve of the source versus the frequency, as well as the measured and simulated gain and aperture efficiency curves of the TA versus frequency.

Figure 12 shows the E-plane and H-plane-normalized radiation patterns of the proposed TA at 24, 27, and 30 GHz. The sidelobe levels were below −20 dB. The cross-polarization levels were also below −20 dB in the broadside direction. The curves of the simulated and measured gain and aperture efficiency of the TA are shown in Figure 13. For a more complete characterization of the proposed metasurface-based TA, it is important to note the gain of the horn antenna used as a source. Therefore, the curve of the simulated gain of the source is also displayed in Figure 13. The value of the simulated gain was from 11.7 to 15.7 dBi within the frequency range of 22 to 32 GHz. The aperture efficiency was calculated using the ratio of the gain to the directivity coefficient of the maximum radiation direction. The differences between the simulated and measured results were mainly caused by the fabrication tolerances and assembly errors. At 29.5 GHz, the antenna had a maximum gain of 24.5 dBi and a maximum aperture efficiency of 41% at 25.5 GHz. The gain variation range of the antenna was less than 1 dB, corresponding to a gain bandwidth of 26.2% (from 23.5 to 30.5 GHz). In addition, the TA achieved a 34% 3-dB gain drop bandwidth (22.5–31.5 GHz).

The comparisons of the proposed wideband TA with previously reported work [2,4,10,11,12] are shown in Table 1. For the TAs in the millimeter-wave, the proposed TA with the MS-based element exhibited the widest 1-dB gain bandwidth of 26.2% compared with the TAs in [2,11,12]. Compared with the TA in [4], the proposed TA has a wider 1-dB gain bandwidth and is easier to integrate with other circuits due to the lack of air gaps. For the TA in [10], it worked in the Ku-band and had a 1-dB gain bandwidth of 37.3%. However, the two air gaps in the element [10] limited its further application. The proposed TA in this work (without air gaps) can be easily integrated with other circuits, which means it is a good candidate for millimeter-wave applications.

## 4. Conclusions

A broadband TA using an MS-based element was presented in this paper, which was composed of 4 × 4 MS as the receiver and transmitter and slot-coupled differentially fed striplines as the phase delay. By designing the size of the sub-patch reasonably, good transmission performance with low insertion loss was achieved. Moreover, a more-than-360° phase shift range within a wide frequency range was achieved by only changing the length of the striplines. The presented TA obtained a 1-dB gain bandwidth of 26.2% and a peak gain of 24.5 dBi, which shows great potential in millimeter-wave communication systems. In addition, the proposed TA is planar and can be conveniently integrated with other planar circuits.

## Figures and Tables

**Table 1:** Performance comparison with previously reported antennas.

Ref.	Frequency (GHz)	Number of Air Gaps	Peak Gain (dBi)	Gain Bandwidth (1-dB)	Gain Bandwidth (3-dB)
[2]	30	2	28.6	7.5%	/
[4]	12	2	25.8	16.8%	/
[10]	15	2	23.06	37.3%	/
[11]	100	None	24.5	/	15%
[12]	20	None	30	9.6%	/
This work	27	None	24.5	26.2%	34%

## Data Availability

The original contributions presented in the study are included in the article. Further inquiries can be directed to the corresponding author.
